# Lipidomic profiling reveals a distinct lipidomic signature of early gestational diabetes

**DOI:** 10.1038/s43856-026-01765-6

**Published:** 2026-07-04

**Authors:** Saeede Saadati, Rasoul Godini, Eveline Jona, Christopher K. Barlow, Adriana CH Neven, Christopher J. Nolan, David Simmons, Helena Teede, Aya Mousa

**Affiliations:** 1 Monash Centre for Health Research and Implementation (MCHRI), Faculty of Medicine, Nursing and Health Sciences, Monash University, Clayton, VIC Australia; 2 Development and Stem Cells Program, Monash Biomedicine Discovery Institute and Department of Anatomy and Developmental Biology, Monash University, Melbourne, VIC Australia; 3 Monash Proteomics & Metabolomics Platform, Department of Biochemistry and Molecular Biology, Biomedicine Discovery Institute, Monash University, Clayton, VIC Australia; 4 Department of Diabetes and Endocrinology, The Canberra Hospital, Canberra, ACT Australia; 5 School of Medicine and Psychology, Australian National University, Canberra, ACT Australia; 6 Macarthur Clinical School, Western Sydney University, Campbelltown, NSW Australia; 7 Robinson Research Institute, Adelaide, SA Australia; 8 Westmead Hospital, Parramatta, NSW Australia; 9 Department of Medicine, Bankstown-Lidcombe Hospital, Bankstown, NSW Australia; 10 Department of Medicine, Blacktown Hospital, Blacktown, NSW Australia; 11 Department of Medicine, Liverpool Hospital, Liverpool, NSW Australia; 12 Nepean Clinical School, Faculty of Medicine and Health, University of Sydney and Nepean Hospital, Penrith, NSW Australia

**Keywords:** Gestational diabetes, Biomarkers

## Abstract

**Background:**

Gestational diabetes mellitus (GDM) diagnosed early in pregnancy (before 20 weeks’ gestation) is associated with increased metabolic risk, yet its molecular profile remains poorly defined. While disrupted lipid metabolism is an important feature of GDM, no previous study has characterized the lipidomic profile of women with early GDM (eGDM).

**Methods:**

We performed untargeted liquid chromatography mass spectrometry on maternal plasma samples from a cohort of 180 women at risk of GDM, enrolled in the Treatment of Booking GDM (TOBOGM) multicenter randomized controlled trial. Oral glucose tolerance tests (OGTT) were conducted before 20 weeks’ gestation to diagnose eGDM using World Health Organization (2013) criteria. Lipidomic data were analyzed using multivariable linear regression, unsupervised clustering, weighted gene co-expression network analysis (WGCNA), and logistic regression-based risk modeling.

**Results:**

In 89 eGDM and 91 non-GDM controls, we quantify 543 lipid species across 18 lipid classes. We identify a distinct lipidomic signature of eGDM, comprising elevated concentrations of glycerolipids (diacylglycerols), fatty acids, and ethanolamine-containing glycerophospholipids (phosphatidylethanolamine and lysophosphatidylethanolamine), alongside lower concentrations of choline-containing glycerophospholipids (lysophosphatidylcholine and ether-linked phosphatidylcholine) and glycosphingolipids (hexosylceramides). Fatty acid and diacylglycerol species are the strongest and most consistent lipid predictors of eGDM and glycemic indices, independent of clinical risk factors. Lipid ontology-based enrichment analysis reveals perturbations in pathways related to lipid storage, membrane remodeling, and signaling.

**Conclusion:**

To our knowledge, this is the first study to characterize the lipidomic profile of women with eGDM. We identify a distinct lipidomic signature associated with eGDM, offering molecular insights into pathophysiology and highlighting candidate lipid biomarkers for future investigation and validation in this context.

## Plain Language Summary

Gestational diabetes can develop early in pregnancy (before 20 weeks) and is associated with a higher risk of complications for both mothers and babies. However, it is not well understood whether patterns of certain molecules in the blood differ between women with early gestational diabetes and those without. In this study, we analyzed blood samples from 180 women at risk of gestational diabetes who underwent early pregnancy glucose testing. Using advanced laboratory techniques to measure a wide range of blood lipids, we identified a distinct lipid pattern in women with early gestational diabetes, characterized by higher levels of several triglycerides and lower levels of specific phospholipids. These findings provide insights into early metabolic changes in gestational diabetes and may inform future research into earlier detection and targeted treatment strategies.

## Abstract (web summary)

Saadati, Godini, et al. perform untargeted lipidomic analysis of maternal plasma samples from 180 women at risk of gestational diabetes. They identify a distinct lipid profile associated with early gestational diabetes, highlighting candidate lipid biomarkers for future investigation and validation.

## Introduction

Gestational diabetes mellitus (GDM) affects approximately 14% of pregnancies worldwide and is characterized by hyperglycemia first identified during pregnancy, with glucose levels less than overt diabetes^1,2^. Early GDM (eGDM), defined as hyperglycemia occurring before 20 weeks’ gestation, accounts for 30–70% of GDM cases^3^. The prevalence of eGDM varies widely across countries, ranging from 1 to 37%, depending on the population, screening protocol, and diagnostic criteria used^4^. Pathophysiologically, eGDM likely reflects pre-existing, subclinical insulin resistance and/or β-cell dysfunction^5^, while GDM developing later (24–28 weeks’ gestation) is driven by responses to the metabolic demands of pregnancy. Metabolic disturbances in eGDM can disrupt placental development during critical early pregnancy stages, thereby impacting both maternal metabolism and fetal growth^6^, and potentially leading to a higher risk of pregnancy complications compared with later-onset GDM^3^.

Disrupted lipid metabolism is increasingly recognized as a central feature in the pathogenesis of eGDM. Lipids serve diverse cellular functions, including energy provision, cell signaling, and membrane structure, and their intracellular homeostasis is dynamically regulated through uptake, de novo synthesis, and hydrolysis^7^. During early pregnancy, insulin secretion increases ahead of the physiological rise in insulin resistance that occurs in mid-to-late pregnancy^6^. Although fatty acids enhance glucose-stimulated insulin secretion, the impact of maternal lipids on islet β-cell function during these early pregnancy stages remains unclear, especially in pregnancies with GDM^6^. Conventional lipid panels in GDM-affected pregnancies reveal an atherogenic pattern, with elevated total cholesterol (TC), low-density lipoprotein cholesterol (LDL-C), and very-low-density lipoprotein cholesterol (VLDL-C), and reduced high-density lipoprotein cholesterol (HDL-C), reflecting dysregulated lipoprotein metabolism, characteristic of cardiometabolic disease states^8,9^. While informative, these panels capture a narrow view of lipid metabolism.

Recent advances in lipidomics mass spectrometry techniques now enable sensitive and simultaneous quantification of hundreds of lipid species implicated in insulin resistance, inflammation, and cellular stress^10^. At this molecular level, subtle alterations in lipid species may precede detectable hyperglycemia on standard oral glucose tolerance tests (OGTTs), offering insights into the metabolic landscape of eGDM and potential avenues for early risk stratification and intervention. Yet, to date, studies have only examined first-trimester lipidomic profiles to predict later GDM^11–15^, most of which were constrained by small sample sizes^14,15^, homogenous ethnic groups (despite known ethnic differences in lipid homeostasis^11,13^), or by examining a narrow subset of lipid species^12^. Lipidomic studies conducted later in pregnancy have yielded highly inconsistent findings and were similarly constrained by small sample sizes^16–23^ and limited ethnic diversity^22,24,25^. To our knowledge, no previous study has characterized the lipidomic profile of women diagnosed with eGDM (i.e., <20 weeks’ gestation) – a clinically important subgroup that appears to be pathophysiologically distinct from later-onset GDM, with higher metabolic risks.

To address this important gap, we aimed to characterize the early pregnancy plasma lipidome in women with eGDM compared with non-GDM controls, and to define candidate lipids of potential biological relevance to eGDM. Our results identify a distinct lipidomic signature associated with eGDM, independent of clinical risk factors, characterized by elevated concentrations of glycerolipids, fatty acids, and ethanolamine-containing glycerophospholipids, alongside lower glycosphingolipids and choline-containing glycerophospholipids. These findings provide insights into potentially disrupted metabolic pathways in eGDM and highlight key lipids for further investigation.

## Materials and methods

### Study design and population

This is an approved sub-study of the Treatment of Booking Gestational Diabetes Mellitus (TOBOGM) multicenter randomized controlled trial, which investigated the identification and treatment of eGDM^26^. Here, we conducted cross-sectional analyses of baseline samples and data from a total of 180 TOBOGM participants from one large site in Australia. All participants from this site with available baseline samples and complete clinical data were included in this sub-study.

In the parent trial, eligible participants were ≥18 years old, with a singleton pregnancy between 4 and 19^+6^ weeks’ gestation and at least one risk factor for hyperglycemia (including previous GDM, body mass index [BMI] >30 kg/m^2^, age ≥40 years, non-European ancestry, polycystic ovary syndrome [PCOS], previous macrosomia, or a first-degree relative with diabetes). A 2-h 75-g OGTT was conducted before 20 weeks’ gestation (between 10 and 19^+6^ weeks’ gestation), and used to diagnose eGDM based on World Health Organization (WHO) 2013 criteria^1^, defined as meeting one or more of: fasting glucose ≥5.1 mmol/L, 1-h glucose ≥10.0 mmol/L, and/or 2-h glucose ≥8.5 mmol/L. Those testing positive at early pregnancy were classified as the eGDM group (irrespective of later GDM status), while the non-GDM group comprised women who tested negative on OGTT both at early pregnancy and at the later routine testing time point (24–28 weeks’ gestation).

### Ethics

This study was conducted as a sub-study of an already approved randomized controlled trial. The parent trial (TOBOGM study) received ethics approval from South-Western Sydney Local Health District Research and Ethics Office, Australia (15/LPOOL/551), the Ethics committee of the Medical University of Vienna, Austria (1337/2016), Swedish Ethical Review Authority, Sweden (2020-05537/2021-00727), and Madras Diabetes Research Foundation Institutional Ethics Committee, India (MDRF/NCT/09-01/2021)^27^. Local approval from the Monash Health Research Ethics Committee (Ref: RES-17-0000-406X) was obtained for recruitment and data collection at the Monash site, which included approval for this sub-study. The trial was registered on 12/07/2016 with the Australian New Zealand Clinical Trials Registry (https://www.anzctr.org.au/, Identifier ACTRN12616000924459) and was conducted in accordance with institutional ethical requirements and regulations. All participants provided written informed consent prior to participation, including for sample collection, storage, and analysis at the Monash site (i.e., bio-banked samples used in the present sub-study).

### Untargeted lipidomic analysis

#### Sample preparation and lipid extraction

Fasting blood samples were collected from participants at the OGTT visit into serum separation tubes and immediately centrifuged, aliquoted, and stored at −80 °C until analysis, with no freeze-thaw cycles to minimize risk of post-sampling lipolysis. Batch lipidomic analysis was then performed at the Monash Proteomics and Metabolomics Platform (Monash University, Melbourne). Samples were thawed once and extracted into 1:1 butanol:methanol using a previously described method^28^, with some modifications. Briefly, in a 1.5 mL microcentrifuge tube, 225 µL of extraction solvent was added to 25 µL of plasma, briefly vortexed, then transferred to a sonic water bath. Ice was added periodically to maintain bath temperature below 25 °C. Samples were then centrifuged at 22,000 × *g*, 20 °C for 10 min. The supernatant was transferred to a mass spectrometry vial with 250 µL glass insert and stored at −80 °C. Immediately prior to analysis, samples were equilibrated to room temperature and sonicated in a water bath for 15 min before transfer to the autosampler for analysis.

The extraction solvent comprised a mixture of 1:1 butanol:methanol with the addition of internal standards corresponding to a commercially available mixture of deuterium labeled lipids, EquiSPLASH™ LIPIDOMIX® Quantitative Mass Spec Internal Standard and 17:1 Lyso-PI (1-(10Z-heptadecenoyl)−2-hydroxy-sn-glycero-3-phospho-(1’-myo-inositol)), ammonium salt (Avanti Polar Lipids, USA). EquiSPLASH contains a mixture of 13 lipids with a deuterium labeled standard in each of the following classes: phosphatidylcholine (PC), phosphatidylethanolamine (PE), phosphatidylserine (PS), phosphatidylglycerol (PG), phosphatidylinositol (PI) and diacylglycerol (DAG), triacylglycerol (TAG), lysophosphatidylcholine (LPC), lysophosphatidylethanolamine (LPE), cholesteryl ester (CE), and monoacylglycerol (MAG), sphingomyelin (SM) and Ceramide (Cer). The PC, PE, PS, PG, PI, and DAG lipids contain 15:0 and 18:1(d7) fatty acids; while LPC, LPE, CE, and MAG contain 18:1(d7) fatty acids; and TAG contains two 15:0 and one 18:1(d7) fatty acids. The Cer standard corresponds to Cer d18:1(d7)/15:0 and the SM standard to SM d18:1/18:1(d9).

These standards were quantitatively transferred to a 250 mL volumetric flask and brought to volume with 1:1 butanol:methanol. To monitor analytical performance, a pooled quality control (PQC) sample was prepared by combining 5 µL from each individual study sample. PQC samples were periodically analyzed throughout the analytical run. Additionally, several PQC samples were extracted as described above, the extracts pooled and then split to generate a series of identical technical quality control (TQC) samples, used to assess extraction and instrument reproducibility.

#### Liquid chromatography mass spectrometry analysis

Liquid chromatography-mass spectrometry (LC-MS) was conducted using a Dionex Ultimate 3000 UHPLC coupled to a Q Exactive Plus mass spectrometer (Thermo Scientific, Australia). Lipid separation was achieved via reversed-phase chromatography on a Zorbax Eclipse Plus C18 Rapid Resolution HD column (2.1 × 100 mm × 1.8 μm, Agilent Technologies, Australia) using a binary solvent system: solvent A (40% isopropanol) and solvent B (98% isopropanol), both containing 2 mM formic acid and 8 mM ammonium formate. The linear gradient for solvent B was as follows: 0 min, 0%; 8 min, 35%; 16 min, 50%; 19 min, 80%; 23 min, 100%; 28 min, 100%; 30 min, 0%; 32 min, 0%. The flow rate was 250 µL min^−1^, column temperature was 50 °C, and injection volume was 10 µL. The mass spectrometer operated at a resolution of 70,000 in polarity switching mode at the MS1 level. PQC samples were also analyzed by data-dependent acquisition in each polarity via separate injections. Here, the MS conditions were the same with the MS1 survey scan from m/z 450 to 1300 (resolution of 70,000) and MS2 performed at a resolution of 17,500 (TopN = 7), with an isolation window of 1.5 m/z, a normalized collision energy of 20, 40, and 60, and dynamic exclusion set to 8.0 s. Electrospray ionization source conditions used for all analyses were: spray voltage 3.5 kV; capillary temperature 300 °C; sheath gas 34; aux gas 13; sweep gas 1; and probe temp 120 °C.

#### Lipid identification and quantification

Putative lipid identification was achieved by MS/MS analysis on PQC samples. Lipids were identified by searching the MS/MS spectra against the LipidBlast^29^ database (v10 Hiroshi Tsugawa fork, modified to include the labeled SPLASH standards) using NIST MSPepSearch^30^. Ion chromatograms corresponding to these putatively identified lipids were extracted using MZmine 2.32^31^, and manually reviewed based on their retention times relative to other species within the same lipid class. The final set of identified lipids was compiled into a database against which all study samples were analyzed to extract the integrated peak area in TraceFinder 4.1 (Thermo Scientific, Australia). The resulting peak area was compared against the corresponding internal standard for that class to provide an approximate concentration. For lipids from classes for which there was no internal standard, a standard from another class was used as specified in Supplementary Data 1. In cases where the same lipid was identified in both positive and negative ionization modes, the polarity yielding the most abundant signal was used for quantification.

#### Data normalization, filtering and nomenclature

To ensure high quality lipidomic data, we excluded species with any missing values across bio-samples (*n* = 77 species; mean missingness 24.17% per lipid) to avoid potential bias introduced by imputation. A further 18 lipid species with a coefficient of variation (CV) > 20% in TQC samples (*n* = 24) were removed to control for technical variability. Notably, 41 of the excluded lipid species with missing values also exhibited high technical variability, suggesting that missingness was associated with low-abundance or less reliably measured lipids. Interparticipant CVs were compared to TQC CVs to ensure no lipid species exhibited lower CVs in the bio-samples than technical replicates. Raw or log10-transformed concentration values were retained for further analysis.

The remaining 543 lipid species belonged to 26 classes, each containing between 1 and 118 members. To facilitate meaningful interpretation and reduce class imbalance, lipid classes with structurally similar or few species were reclassified, resulting in 18 broader classes. Members of ceramide α-hydroxy fatty acid-dihydrosphingosine (Cer-ADS), ceramide α-hydroxy fatty acid-phytosphingosine (Cer-AP), ceramide non-hydroxy fatty acid-dihydrosphingosine (Cer-NDS), and ceramide non-hydroxy fatty acid-sphingosine (Cer-NS) were combined into the class “ceramides” (Cer). Additionally, six classes (glucuronosyl diacylglycerol (GlcADG), lysophosphatidic acid (LPA), oxidized phosphatidylcholine (OxPC), oxidized phosphatidylglycerol (OxPG), PG, sulfatide hexosylceramide (SHexCer)) with two or fewer species were grouped into the “other” class. To simplify lipid species nomenclature throughout the manuscript, the sum composition of carbon atoms and double bonds was used as a naming convention by LIPID MAPS^32^. Detailed names and characteristics for all lipid species analyzed in this study are provided in Supplementary Data 1.

### Statistical analyses

#### Principal component analysis

Principal component analysis (PCA) was performed using the *FactoMineR* package, incorporating all 543 included lipid species. Log-transformed lipid abundance values were standardized by z-score normalization prior to analysis. The first five principal components (PCs) (out of 179) accounted for more than 65% of the total variance and were therefore retained. PC1 and PC2 were used for visualization purposes. Statistical comparisons between the distributions of non-GDM and eGDM data points were performed using a two-sided *t* test based on their eigenvectors.

#### Correlation analyses

Correlation analyses were performed using the *rcorr* function from the *Hmisc* package^33^ or *cor.test* function from the *stats* package in R. Both Pearson and Spearman methods were applied, depending on the nature of the data. In scatter plots with density information, the distribution of data points was shown as density with bins = 7. Results were visualized using the *ggplot2* package in R.

#### Unsupervised clustering of samples

Unsupervised clustering was performed using the “*k*-means” method (1000 repeats and based on Euclidean distance) to identify distinct lipid classes and species based on their log10-transformed and standardized levels. The optimal number of clusters (*k*) was calculated using the “within-cluster sums of squares” method^34^. Cluster stability was assessed using the Jaccard bootstrap method with 1000 iterations, using the *fpc* package in R. Results were visualized using the *ComplexHeatmap* package in R^35^.

#### Network analysis based on WGCNA

As has previously been done to analyze lipidome data^36,37^, we applied Weighted Gene Co-expression Network Analysis (WGCNA) to construct a network of lipid species interactions and identify lipid species clusters (modules) associated with eGDM and other clinical characteristics. These modules represent groups of pairwise correlated lipid species that can be related to clinical phenotypes. An optimal soft threshold of 14 and a “signed” network type were used to calculate the adjacency matrix, followed by transformation into a topological overlap matrix. Modules of co-expressed lipids were identified using the *cutreeDynamic* function, with a minimum size of 20 and deepSplit = 3. Modules were considered significant if they showed a minimum correlation of 0.2 and a *P* < 0.01 with clinical characteristics.

Lipid class enrichment within each module was analyzed using a one-tailed Fisher’s exact test^38^. Adjusted *P* values were calculated using the *p.adjust* function from the *stats* package (version 3.6.2) with the Benjamini-Hochberg method^39^. Interaction networks were filtered to include edges with weights >0.3 in module 1 and >0.1 in modules 5 and 8. Centrality was calculated based on weighted degree values. Plots were visualized in *Cytoscape* (V3.10.3)^40^ and *ggplot2* in R.

#### Lipid functional enrichment analysis

We used the LION database (lipidontology.com) to perform lipid functional enrichment analysis^41^. The analysis employed the “target-list mode” approach. Enrichment was assessed using a one-tailed Fisher’s exact test, and terms with a false discovery rate (FDR) < 0.05 were considered significant.

#### Linear regression analysis

All linear regression analyses were performed using the *lm* function from *stats* package (V3.6.2) in R. Lipid species data were log10-transformed prior to modeling. For lipid class analyses, concentrations of lipid species within each class were summed and log10-transformed before modeling. Five models were constructed, adjusted for relevant covariates, as follows. Model 1 was unadjusted. Model 2 was adjusted for maternal age, BMI, gravidity, gestational week at sampling, ethnicity, and family history of diabetes - factors known to influence both lipid metabolism and the risk of eGDM^42^. Pre-pregnancy BMI was not consistently available; thus, BMI at 10–19 weeks’ gestation was used as a proxy for baseline maternal adiposity. Model 3 was further adjusted for maternal educational qualifications, as an indicator of socioeconomic status influencing diet, lifestyle, and overall health^43^. Model 4 was additionally adjusted for neonatal sex, given the sexual dimorphic alterations that influence placental function, lipid composition, and maternal glucose metabolism during pregnancy^44^. Model 5 extended Model 2 with further adjustment for PCOS, previous GDM, and systolic blood pressure (SBP). PCOS and previous GDM are important confounders of eGDM, and SBP differed by eGDM status in this cohort (Table 1). In addition, linear regression analysis was performed to assess the association between lipidomics profiles and differences in age, BMI, and gestational week at sampling to assess within and between group changes in non-GDM and eGDM participants. These models were adjusted for gravidity, ethnicity, and family history of diabetes. Age, BMI, and gestational week at sampling were included as covariates when these variables were not the primary predictors. Multicollinearity was assessed by calculating variance inflation factors (VIF), with predictors retained if VIF was ≤ 4, using the *car* package (V3.1–3)^45^ in R. Percentage difference in lipid species concentrations in eGDM compared with non-GDM controls were calculated using the formula^46^:

$${{{\rm{Difference}}}} \% =\left({10}^{{{{\rm{\beta }}}}{{{\rm{coefficient}}}}}-1\right)\times 100$$

**Table 1:** Sample characteristics at early pregnancy for overall cohort and by eGDM status

Characteristics	All (*n* = 180)	eGDM (*n* = 89)	Non-GDM (*n* = 91)	*P*
Age at first trimester (years)	33.14 ± 4.54	33.96 ± 4.21	32.34 ± 4.74	**0.016**
Ethnicity, n (%) Caucasian South Asian East Asian Southeast Asian Black African Middle Eastern Other	41 (22.78) 81 (45) 20 (11.11) 19 (10.56) 1 (0.56) 3 (1.67) 15 (8.34)	19 (21.35) 39 (43.82) 12 (13.48) 11 (12.36) 0(0) 1 (1.12) 7 (7.87)	22 (24.18) 42 (46.15) 8 (8.79) 8 (8.79) 1 (1.10) 2 (2.20) 8 (8.79)	0.826
Qualification, n (%) No formal qualification Certificate/ diploma Trade/ apprenticeship School/ intermediate Higher school University degree	1 (0.56) 35 (19.44) 6 (3.33) 3 (1.67) 10 (5.56) 122 (67.78)	0(0) 17 (19.10) 3 (3.37) 2 (2.25) 8 (8.99) 56 (62.92)	1 (1.10) 18 (19.78) 3 (3.30) 1 (1.10) 2 (2.20) 66 (72.53)	0.188
Previous GDM, n (%)	33 (18.33)	23 (25.84)	10 (10.99)	**0.028**
Family history of diabetes, n (%)	86 (47.78)	41 (46.07)	45 (49.45)	0.901
PCOS, n (%)	40 (22.22)	27 (30.34)	13 (14.29)	**0.010**
Gravidity, n (%) Primigravida 2−3 4–5 >5	50 (27.93) 91 (50.84) 26 (14.53) 12 (6.70)	18 (20.22) 51 (57.30) 12 (13.48) 8 (8.99)	32 (35.56) 40 (44.44) 14 (15.56) 4 (4.44)	0.081
Parity, n (%) Nulliparous Para 1 Para 2−3 Para >3	69 (38.33) 75 (41.67) 32 (17.78) 4 (2.22)	28 (31.46) 42 (47.19) 16 (17.98) 3 (3.37)	41 (45.05) 33 (36.26) 16 (17.58) 1 (1.10)	0.212
Gestational age at sampling	15.94 ± 2.40	15.85 ± 2.43	16.02 ± 2.38	0.640
Gestational age categories (weeks), n (%) 10–13 weeks’ gestation 14–15 weeks’ gestation 16–17 weeks’ gestation 18–19 weeks’ gestation	28 (15.56) 47 (26.11) 39 (21.67) 66 (36.67)	14 (15.73) 26 (29.21) 16 (17.98) 33 (37.08)	14 (15.38) 21 (23.08) 23 (25.27) 33 (36.26)	0.622
Weight at sampling (kg)	74.71 ± 23.84	79.68 ± 27.86	69.73 ± 17.80	**0.005**
BMI at sampling (kg/m^2^)	28.49 ± 8.14	30.41 ± 9.29	26.58 ± 6.30	**0.001**
SBP at sampling (mmHg)	110.98 ± 11.72	113.82 ± 11.37	108.25 ± 11.47	**0.001**
DBP at sampling (mmHg)	67.88 ± 9.48	69.25 ± 9.89	66.55 ± 8.93	0.061
Neonatal sex, n (%) Male Female	86 (47.78) 91 (50.56)	43 (48.31) 45 (50.56)	43 (47.25) 46 (50.55)	0.851

Continuous variables are presented as mean±SD if normally distributed, or median (IQR) if non-normally distributed. Categorical variables are presented as n (%). P = differences based on chi-square tests for categorical variables and independent samples *t* tests for continuous variables, with log transformation applied prior to analysis for non-normally distributed variables.

*GDM* gestational diabetes mellitus, *PCOS* polycystic ovary syndrome, *BMI* body mass index, *SBP* systolic blood pressure, *DBP* diastolic blood pressure, *n* number, *SD* standard deviation.

The 95% confidence intervals were converted accordingly. *P* values were adjusted using the *p.adjust* function from the *stats* package (version 3.6.2) with the Benjamini-Hochberg method^39^. Lipid species were considered significantly different if the adjusted *P* value was <0.05. Results were visualized using the *ggplot2* package in R.

## Results

### Sample characteristics

**Fig. 1:**
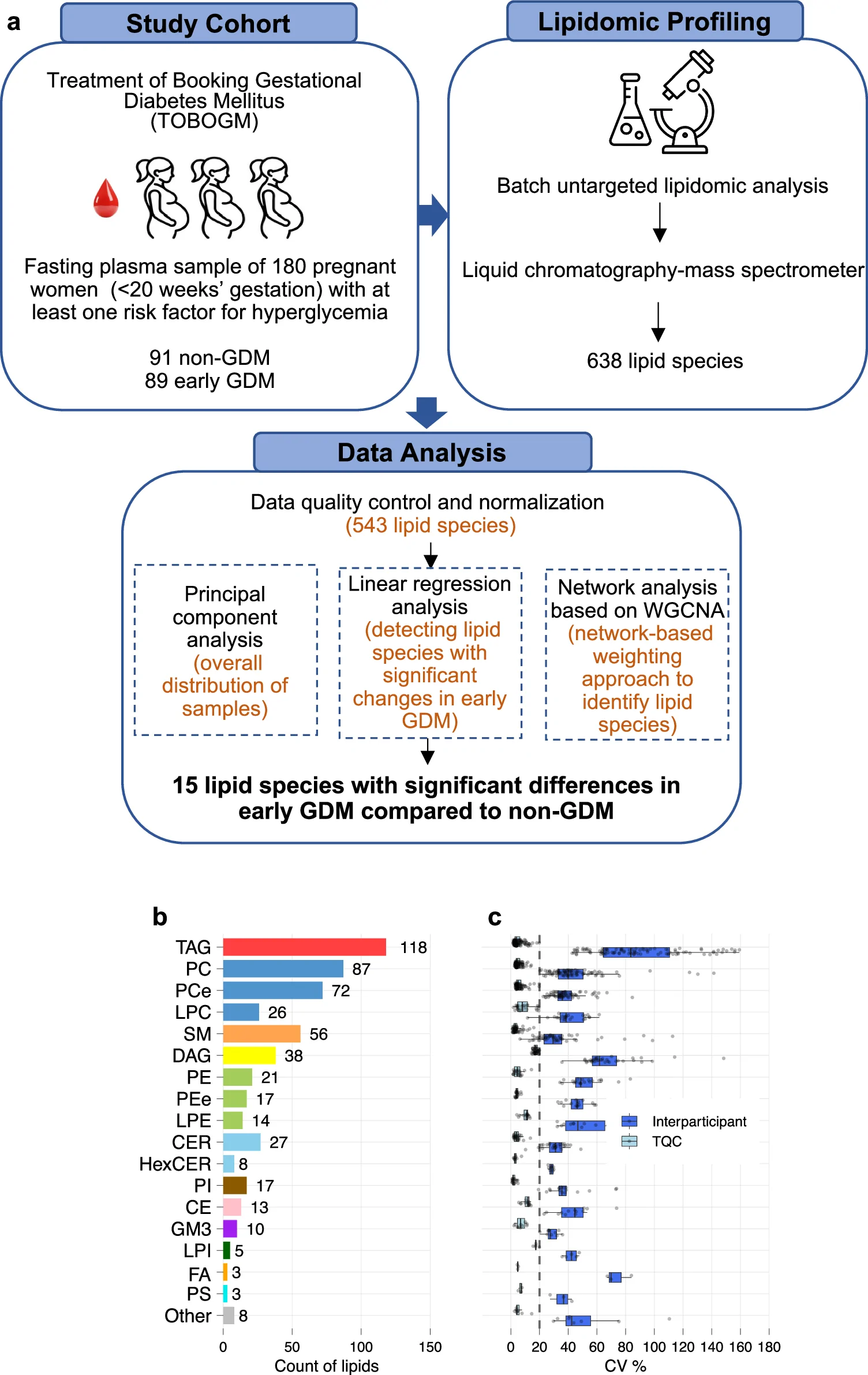
Experimental design and descriptive analyses of clinical and lipidomic data. **a** Schematic of the analytical workflow of the study. As a sub-study of Treatment of Booking Gestational Diabetes Mellitus (TOBOGM), blood samples from 180 participants with at least one risk factor for hyperglycemia were used to perform plasma lipidomic analysis. Batch lipidomic analysis was conducted using liquid chromatography–mass spectrometry (LC-MS), identifying 638 lipid species across all samples. Following quality control assessment to remove low-quality data, 543 lipid species were retained for downstream statistical analysis. Multiple linear regression modeling identified 17 lipid species with statistically significant differences between non-GDM and eGDM samples. **b** A bar chart illustrates the number of lipid species detected across 18 lipid classes. The “other” category represents a lipid class with two or fewer lipid species. Lipid species with missing data or a coefficient of variation (CV) > 20% were excluded, resulting in 543 lipid species. **c** CV analysis showed <20% variation in Technical Quality Control (TQC) samples, while higher variation was observed in certain lipid classes across interparticipant samples.

An overview of the analytical workflow is provided to clarify the sequence and rationale of the study design and analytical steps (Fig. 1a). Of the 180 participants, 89 were diagnosed with eGDM based on WHO 2013 criteria^1^, and 91 were non-GDM controls. Participant characteristics are presented in Table 1. All women were sampled between 10 and 19^+6^ weeks’ gestation, with the mode at week 18 (*n* = 41) and no differences in time of sampling between groups. Those with eGDM had higher maternal age (*P* = 0.016) and BMI (*P* = 0.001) compared to the non-GDM group. Ethnic distribution was diverse, with the highest representation from South Asian (*n* = 81, 45.0%), Caucasian (*n* = 41, 22.8%), East Asian (*n* = 20, 11.1%), and Southeast Asian (*n* = 19, 10.6%) ethnicities, and no differences between groups. Gravidity, parity, and family history of diabetes were similar between eGDM and non-GDM groups, whereas women with eGDM were more likely to have a history of previous GDM and PCOS (Table 1).

### Overall lipidome profile of participants

The primary lipidome data consisted of 638 lipid species across 180 samples. Following removal of lipid species with missing data or high variation (CV > 20%) in TQC samples, 543 lipid species were included in the analysis (Supplementary Data 1). Across these 543 lipid species and classes (Fig. 1b), technical reproducibility was high, with CVs ≤ 20% in TQC samples and greater interparticipant variation observed in the fatty acid (FA), TAG, and DAG classes (Fig. 1c). Lipid species were categorized into 18 classes, with the highest numbers of species in TAG (*n* = 118) and PC (*n* = 87) and the lowest in FA, lysophosphatidylinositol (LPI), and PS (each with *n* = 3–5) (Fig. 1b; Supplementary Data 1).

### Lipid variation by eGDM status on principal component analysis

**Fig. 2:**
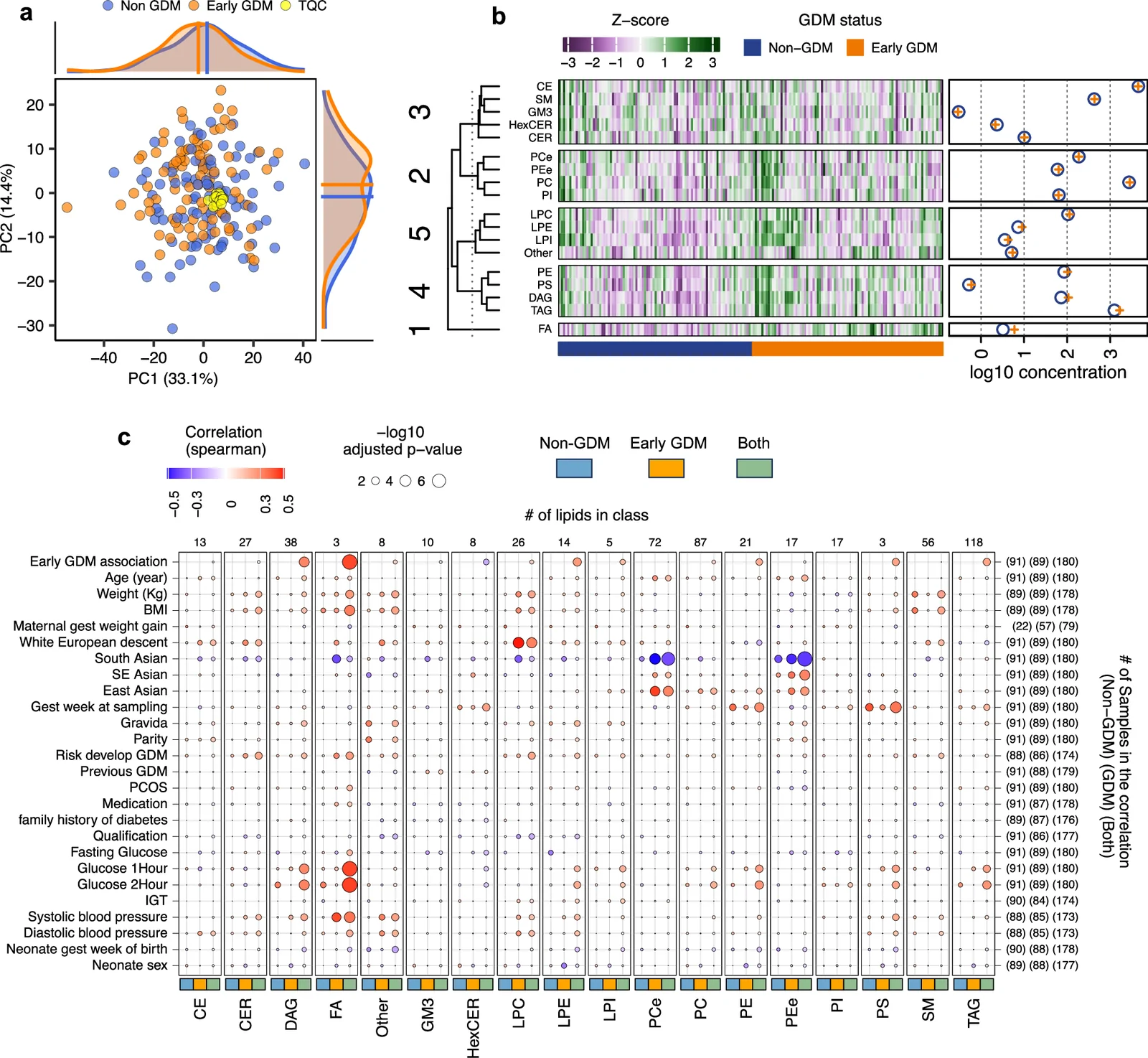
Lipidomic profile variation and correlation analyses between non-GDM and eGDM participants. **a**Principal Component Analysis (PCA) of eGDM and non-GDM samples shows variation based on 543 lipid species. Histograms highlight significant differences between the two groups in both PC1 and PC2, with greater distinction observed in PC2 (*P*_*t-test*_ = 0.004). Yellow dots indicate TQC samples, which cluster tightly, supporting the absence of systematic drift or batch effects during the analytical run. **b** Heatmap of standardized log10 lipid concentrations across lipid classes. Unsupervised clustering identified five distinct clusters based on dissimilarity. The annotation on the right compares median log10 scaled peak area for each class, represented by blue circles (non-GDM) and orange plus signs (eGDM). The values used in the heatmap are listed in Supplementary Data 2. **c** Correlation analyses between lipid classes and clinical characteristics were conducted using the Spearman method, with no imputation or normalization applied to clinical data. Analyses were performed separately for non-GDM and eGDM groups, as well as collectively, to capture underlying associations. The numbers above indicate the count of lipids in each class, while the numbers on the right denote the sample size for each group (non-GDM, eGDM, and combined). The size of each dot indicates significance reported as log10 of adjusted *P* values (Benjamini-Hochberg method). Information used in the correlation plots is listed in Supplementary Data 2.

PCA was conducted as an exploratory analysis to assess the overall structure of the lipidomics data and to evaluate whether global lipid variation separated samples according to clinical status. The results showed overlapping sample distributions in the 543 included lipid species measured, indicating no global or distinct difference between the eGDM and non-GDM groups (Fig. 2a). When assessing the density of samples from each group, differences were seen across principal components 1 (*P* = 0.046) and 2 (*P* = 0.004) between groups (Fig. 2a). Eigenvector values of each point across principal components are listed in Supplementary Data 2.

To explore differences between groups at the lipid class level, unsupervised clustering was conducted on the log10-transformed sum of all lipid species within each class (Fig. 2b; Supplementary Data 2). Five distinct clusters of lipid classes were identified. Cluster-wise Jaccard bootstrap mean values were as follows: cluster 1: 0.53; cluster 2: 0.651; cluster 3: 0.781; cluster 4: 0.636; and cluster 5: 0.834, indicating high to moderate stable clusters. Among these, Cluster 1, which contains only the FA class, and Cluster 4 comprising PE, PS, DAG, and TAG, showed higher lipid concentrations in participants with eGDM (Fig. 2b). Cluster 5 includes lipid classes such as LPE and LPI, which exhibited some differences, while LPC and “other” lipids showed no differences between eGDM and non-GDM groups. Clusters 2 and 3 demonstrated no significant difference between groups. These findings suggest a potential association of the FA, PE, PS, LPE, LPI, DAG, and TAG lipid classes with the presence of eGDM.

### Correlation analysis of lipid class variation by eGDM status

Correlation analyses between lipid classes and eGDM, as well as clinical risk factors for GDM, were performed in the combined cohort and separately for each group (Fig. 2c; Supplementary Data 2). Associations with other clinical risk factors are detailed in Supplementary Data 3, with a summary of results provided in Supplementary Information; Supplementary Fig. S1a–f. Across the cohort, the strongest correlations with eGDM were observed for FA (*r* = 0.44, *P* = 4.4 × 10^−10^), DAG (*r* = 0.32, *P* = 1.3 × 10^–5^), and LPE (*r* = 0.27, *P* = 2.6 × 10^–4^). Weaker correlations were seen for TAG (*r* = 0.24, *P* = 1.1 × 10^–3^), PS (*r* = 0.24, *P* = 1.4 × 10^–3^), PE (*r* = 0.22, *P* = 3.1 × 10^–3^), and LPI (*r* = 0.18, *P* = 0.017), while hexosylceramides (HexCer) were the only class negatively correlated with eGDM (*r* = –0.19, *P* = 0.011).

FA and DAG were also highly positively correlated with 1- and 2-h glucose concentrations, with similar coefficients (Supplementary Data 2), while only FA was associated positively with fasting blood glucose (*r* = 0.19, *P* = 0.010) and HexCer associated negatively (*r* = –0.16, *P* = 0.032; Fig. 2c). Other lipid classes, including LPE, LPI, TAG, PS, PE, and PC showed weaker, but statistically significant, correlations with 1- and 2-h glucose levels. Together, both PCA and correlation analyses suggest a lipidomic signature for eGDM comprising higher glycerolipids (DAG, TAG), glycerophospholipids (PE, PS, LPE, LPI), and FA, and lower glycosphingolipids (HexCer), with FA and DAG showing the strongest correlations with blood glucose concentrations.

### Linear regression analysis of differential lipid species by eGDM status

**Fig. 3:**
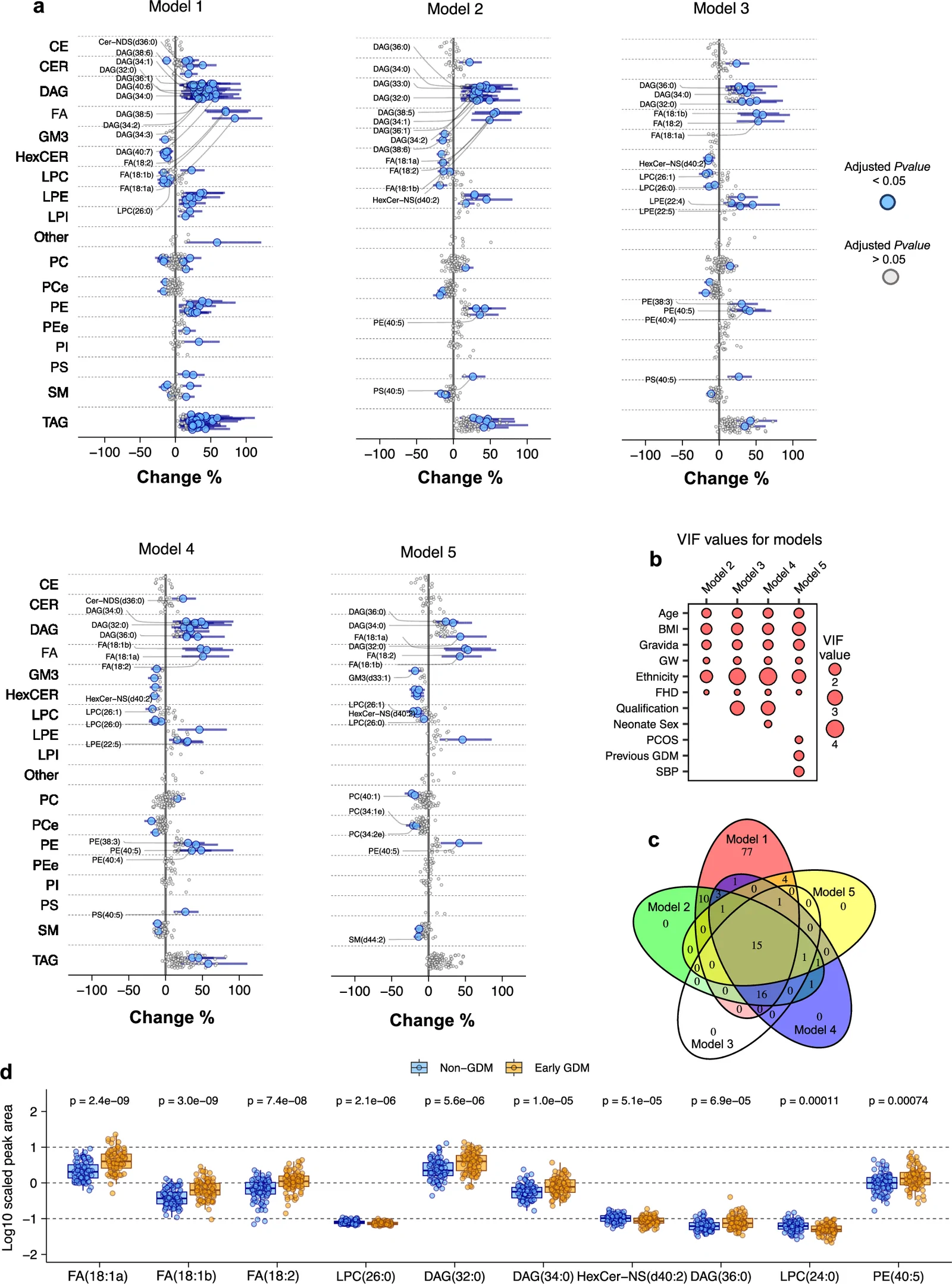
Linear regression analysis identifying associations between lipidomic profiles and eGDM incidence. **a** Linear regression analyses were performed to examine percentage change in lipid species concentrations in eGDM compared to non-GDM controls under four models. Model 1: unadjusted, associating lipid species levels with eGDM presence (*n* = 180); Model 2: adjusted for maternal age, BMI, gravidity, gestational week at sampling, ethnicity, and family history of diabetes (*n* = 178); Model 3: Model 2 plus adjustment for educational qualification (*n* = 172); Model 4: Model 3 plus adjustment for neonatal sex (*n* = 172); and Model 5: Model 2 plus adjustment for PCOS, previous GDM and systolic blood pressure (SBP) (*n* = 167). Multicollinearity was assessed with a threshold variation inflation factor (VIF) of 4. Predictors included maternal age, BMI, gestational week at sampling, and gravidity as continuous variables, while ethnicity, qualification, family history of diabetes, and neonatal sex were included as binary variables. *P* values were corrected for multiple testing using the Benjamini-Hochberg method. The measure of center represents the percentage change (back-transformed from the log10 scale), and the error bars represent the 95% confidence intervals (also back-transformed from the log10 scale). **b** Bubble plot showing the VIF values of the confounders across different linear regression models. **c** Venn diagram showing the overlap of significant lipid species across the five linear regression models. **d** The top 10 most significant lipid species across all linear regression models, presented as crude independent samples *t-*tests for visualization purposes. The lipid peak areas are scaled to the peak area and molar amount of the corresponding internal standard of the same class, or in the case of the FAs the PE internal standard. FA (18:1a) refers to FA (18:1 [2M-H]); and FA (18:1b) refers to FA (18:1 [2 M+Na-2H]).

To identify individual lipid species which differ by eGDM status, five linear regression models were constructed, using lipid species concentrations as the dependent variable, with eGDM status as the primary exposure, adjusted for relevant covariates (Fig. 3a). The VIF results showed there was no multicollinearity between the predictors (Fig. 3b; Supplementary Data 4). The full list of lipid species identified by the models and related information are provided in Supplementary Data 4. Consistent with the lipid class results (Fig. 2b-c), most lipid species across all models showed higher concentrations in the eGDM compared to the non-GDM group. In the unadjusted model (Model 1), we identified 128 lipid species with significantly different levels between the groups across 16 of the 18 lipid classes. With the inclusion of covariate predictors, the number of significant lipid species decreased to 48 in Model 2, 33 in Model 3, 40 in Model 4, and 23 in Model 5. This attenuation suggests that these factors may partly explain the lipid differences observed between eGDM and non-GDM in earlier models. However, a subset of lipid species remained significant across all models, indicating some robust associations independent of measured confounders. Across the models, lipid species from the Cer, DAG, FA, LPE, PE, and PS classes showed significantly higher concentrations in the eGDM group, whereas those from the ganglioside GM3, HexCer, SM, LPC, PC and PCe classes showed relatively lower concentrations.

The models showed considerable overlap, with 15 lipid species from classes of FA, DAG, PE, LPE, HexCer, LPC, and PCe identified as significant across all five models (Fig. 3c; Supplementary Data 4). These 15 overlapping lipid species were further clustered to explore associations between peak area and clinical characteristics included in the models (Fig. 3d).

To further compare the identified lipid species between the early and non-GDM groups, we conducted *t-tests* on the overlapping lipid species and visualized the top 10 results (Fig. 3d; Supplementary Data 4). Species from the DAG, FA, LPC and HexCer classes had the lowest *P* values compared to others, aligning with the results of the linear regression analyses. Together, multivariable results suggest that lipid species from the FA, DAG, LPE and PE classes exhibit higher concentrations, while LPC, PCe and HexCer showed lower concentrations in the eGDM group. Among these, FA and DAG species represent the most significantly altered lipid species between early and non-GDM groups.

### Lipid clusters associated with eGDM using WGCNA

**Fig. 4:**
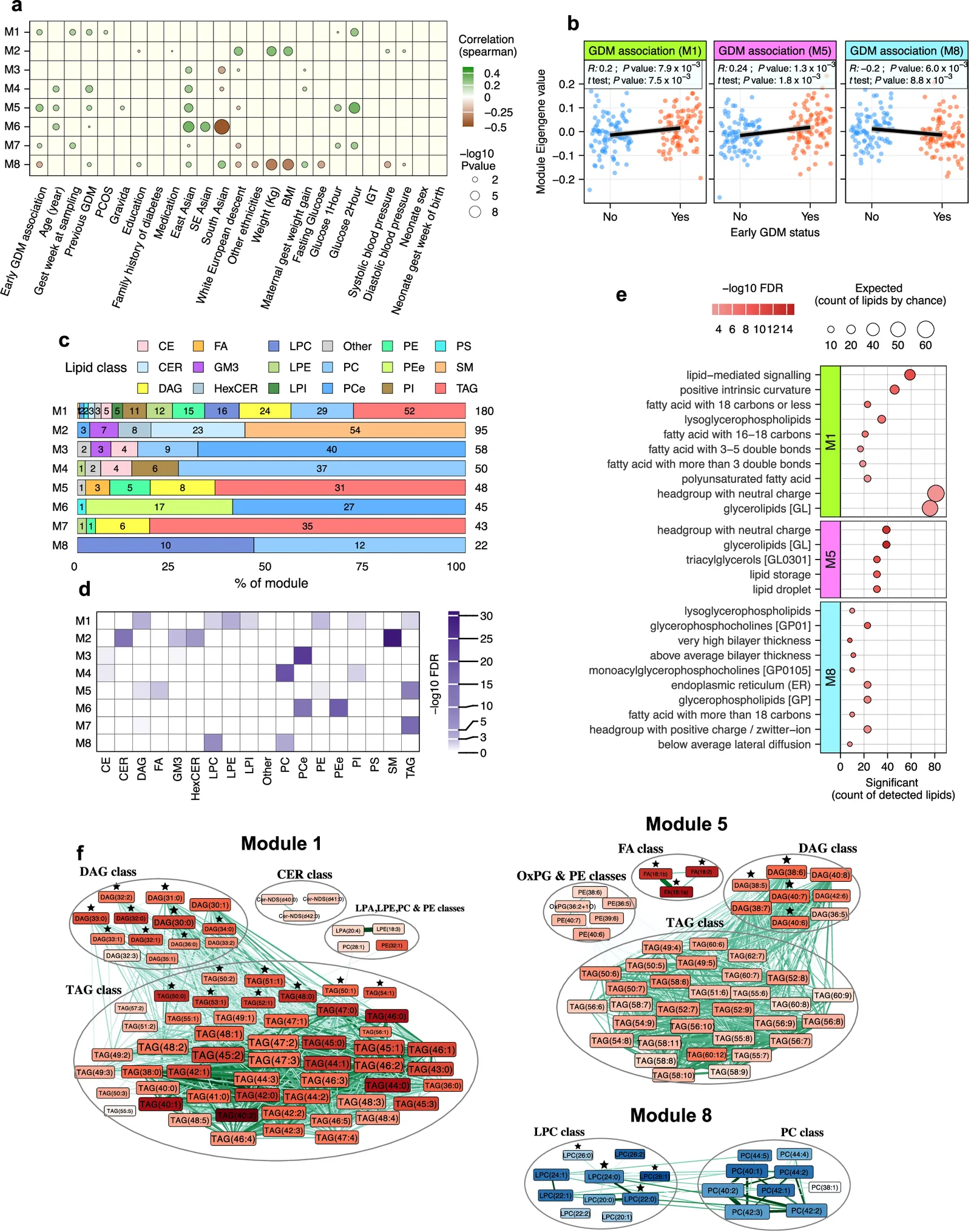
Lipidomic profile analysis using Weighted Gene Co-expression Network Analysis (WGCNA). **a** WGCNA identified eight modules based on 543 lipid species and their associations with clinical characteristics. **b** Correlation and *t-*test analyses of eigengene values from candidate modules between non-GDM and eGDM samples revealed significant associations for modules 1, 5, and 8 with eGDM. The *t-test* results (two-sided and unadjusted) supported the correlation findings. **c** The distribution of lipid classes within each module is presented as a stacked bar chart, showing the percentage of each class. Numbers within stacks indicate lipid counts, while numbers on the left denote total lipids in each class. **d** Enrichment analysis of lipid classes within each module was performed using Fisher’s exact test. Results are displayed as a heatmap of -log10 false discovery rate (FDR) values, indicating significant enrichment. **e** Lipid ontology analysis of module members was performed using the LION (lipidontology.com/) database. The top terms with adjusted *P* < 0.05 (up to 10 terms) are shown, highlighting processes and terms enriched by the lipids. Significance is reported as -log10 of adjusted *P* values (Benjamini-Hochberg method), comparing detected lipid counts with expected counts. **f** Networks of the candidate modules were constructed using WGCNA. Networks were filtered to retain strong interactions (M1 ≥ 0.3, and M5/M8 ≥ 0.1). Lipid classes within each network are grouped, with larger nodes representing lipids with more and stronger interactions. Star symbols denote lipids with significant expression differences between non-GDM and eGDM samples based on linear modeling (Model 2: adjusted for maternal age, BMI, gravidity, gestational week at sampling, and ethnicity). Node colors represent expression changes (red: positive; blue: negative), with intensity indicating the magnitude of alteration. Thicker, darker edges indicate stronger interactions. FA (18:1a) refers to FA (18:1 [2M-H]); and FA (18:1b) refers to FA (18:1 [2 M+Na-2H]).

We applied WGCNA to identify lipid species clusters associated with eGDM and other clinical characteristics of participants. By considering scale-free topology fit results (Supplementary Information; Supplementary Fig. S2), a network was constructed using a soft threshold of 14, with eight modules (sub-networks) associated with clinical data of participants. Of these, modules 1, 5, and 8 were significantly correlated (*r* ≥ 0.2) with eGDM (Fig. 4a, b; Supplementary Data 5). Module 1, the largest module, contained 180 lipid species from various classes, including 52 from the TAG class. Module 5 included 48 lipid species enriched primarily from the TAG and FA classes. Module 8 comprised 22 lipid species predominantly from the LPC and PC classes (Fig. 4c). Following enrichment of lipid classes in each module using Fisher’s exact test (Fig. 4d) and annotation using the LION database^41^ (Fig. 4e), module 1 was associated with lipid-mediated signaling and various fatty acids; module 5 was related to glycerolipids and triacylglycerols, lipid storage, and droplet; and module 8 was associated with diverse terms, including glycerophosphocholines, glycerophospholipids, endoplasmic reticulum, bilayer thickness, and lateral diffusion. The complete list of statistically significant terms is presented in Supplementary Data 5. Each module comprised lipid species represented as nodes connected to one another, with hub (central) nodes representing the most strongly connected species. Hub species were identified using network analysis (Fig. 4f) and those significantly different between early and non-GDM groups were annotated for validation with the linear regression analysis results. In module 1, several species from the DAG and TAG classes, such as DAG (30:0), (31:0), (32:0), (33:0), and TAG (48:0) and (51:1) were highly connected and significantly over-expressed in the eGDM group. In module 5, hub species included DAG species (DAG (38:5) (38:6) (40:6) (40:7)) were also significantly higher in eGDM. Hub species in module 8 belonged to the PC and LPC classes, with LPC (22:0), (24:0), (26:0), and (26:1) showing lower concentrations in the eGDM group (Fig. 4f).

Overall, the three lipid modules associated with eGDM in WGCNA included hub species overlapping those identified through linear regression modeling, indicating consistent associations across analytical approaches.

## Discussion

To our knowledge, this is the first study to characterize the plasma lipidome of women with eGDM (diagnosed before 20 weeks’ gestation). Using untargeted lipidomic profiling, we quantified 543 lipid species (18 lipid classes) in 180 women during early pregnancy, including 89 with eGDM. PCA demonstrated only subtle global differences in the lipidome profile of eGDM compared with non-GDM groups, likely due to the high inter-individual variability in lipid concentrations across the overall cohort. However, deeper analyses across multiple complementary statistical approaches, including unsupervised clustering, correlation analyses, regression modeling, and WGCNA, revealed consistent differences at the lipid class and species levels, identifying a distinct lipidomic signature associated with eGDM. This lipidomic signature was marked by elevated concentrations of glycerolipids (DAG), FA, and ethanolamine-containing glycerophospholipids (PE and LPE), alongside lower concentrations of glycosphingolipids (HexCer) and choline-containing glycerophospholipids (LPC and PCe). Among these, FA and DAG were the most consistently and positively associated with eGDM and correlated with dysglycemia, while HexCer exhibited a consistent negative association, suggesting these lipids may have potential relevance as early markers for eGDM. In WGCNA, modules enriched for FA, DAG and TAG species (Modules 1 and 5) were significantly associated with eGDM and were annotated for functions related to glycerolipid metabolism, lipid storage, and lipid-mediated signaling, underscoring the coordinated disruption of metabolic pathways in eGDM.

FAs, oleic acid and linoleic acid, which exhibited the strongest correlations with eGDM and with fasting, 1-h, and 2-h blood glucose concentrations, belong to the families of monounsaturated and polyunsaturated fatty acids, respectively^47,48^. Oleic acid is largely synthesized in the liver and adipose tissue, esterified in TAGs, and transported via LDL-C and VLDL-C^49^. While generally considered metabolically beneficial, some evidence suggests that, in insulin-resistant states, elevated oleic acid may also be linked to endoplasmic reticulum stress and inflammation^50^. A recent lipidomics study reported higher plasma esterified oleic acid in women with GDM at 24–28 weeks requiring pharmacological treatment compared with those managed by diet alone, suggesting it may be a marker of GDM severity later in pregnancy^51^. Here, we extend this evidence by identifying elevated oleic acid concentrations much earlier in pregnancy, potentially reflecting early lipid dysregulation, though its functional role remains unclear. Similarly, linoleic acid, the parent FA of the n-6 polyunsaturated fatty acid family usually associated with improved insulin sensitivity and metabolic health^52–54^, was also elevated in eGDM. This could reflect impaired conversion of linoleic acid to downstream metabolites (e.g., arachidonic and adrenic acids), and thus altered metabolite ratios, as has been reported in previous studies^55^. Such patterns are consistent with a functional deficit in desaturase and elongase activity and consequent linoleic acid accumulation. Together, our findings of consistent and robust elevation of FAs across correlation and network analyses point to disrupted FA metabolism in eGDM rather than protective effects, highlighting the potentially important but incompletely understood role of FAs in early gestational metabolic regulation.

DAGs were similarly among the most strongly and consistently elevated lipids in eGDM, showing positive associations with post-load glycemia and consistent enrichment in co-expression modules. Elevated DAGs have been previously observed in women with GDM and obesity^56^, though not in the context of eGDM. As key intermediates in glycerolipid synthesis, DAGs impair insulin signaling through activation of protein kinase C isoforms, particularly in insulin-sensitive hepatic and adipose tissues^57^. This pattern aligns with increased de novo lipogenesis – a process known to be activated in insulin-resistant states and implicated in the pathogenesis of GDM^58^. Here, TAGs were also elevated in eGDM, though less consistently, and were co-localized with DAGs in modules related to lipid storage and metabolic signaling. These elevations likely reflect excess lipid flux and diminished oxidative capacity—hallmarks of a lipotoxic metabolic environment^59^. Together, the upregulation of DAGs and TAGs likely reflects contemporaneous lipid accumulation and insulin resistance in early pregnancy in women with eGDM.

Several glycerophospholipid classes, including PE, PS, LPE, and LPI, were elevated in eGDM, though with more variable significance across analyses. Glycerophospholipids were previously reported to be the most prevalent altered lipid species in a small cohort of women with later GDM (*n* = 49), compared with non-GDM (*n* = 54)^24^. PE, the second most abundant phospholipid in cell membranes, is closely associated with insulin resistance and can be enzymatically converted into TAG through the Kennedy pathway – a key metabolic route for phospholipid synthesis^60^. This diversion of excess PE into TAG may promote ectopic lipid accumulation, lipotoxicity, and impaired insulin signaling^60^, suggesting a potential mechanistic link between phospholipid remodeling and metabolic dysfunction in early pregnancy^13^. PS and LPE also showed moderate associations with eGDM, potentially reflecting increased membrane turnover or apoptotic signaling. LPI, a bioactive lysophospholipid and GPR55 agonist^61^, was modestly but significantly elevated in eGDM, reflecting possible hyperinsulinemia^62^. We have previously reported that LPI may stimulate insulin secretion in individuals with obesity^63^ or PCOS^64^, substantiating the insulinotropic effects of LPI seen in early animal models^65^. Notably, other phospholipid subclasses, including LPC and PCe, were consistently downregulated in eGDM. LPC is involved in HDL metabolism and lipid signaling, while PCe (plasmalogen-PCs) possess antioxidant properties; thus, their reduction may reflect impaired lipid remodeling or increased oxidative stress in eGDM^66^. Together, the concurrent up- and downregulation of distinct phospholipid species suggests broader perturbations in both structural and signaling lipid pathways that may be associated with the metabolic phenotype of eGDM.

Beyond these core lipid classes, differences in sphingolipid metabolism were also evident in eGDM. Ceramide species were elevated across multivariable models, aligning with their well-established role in promoting insulin resistance, inflammation, and β-cell dysfunction through inhibition of Akt signaling and activation of pro-apoptotic pathways^67,68^. In contrast, glycosphingolipids including HexCer and, to a lesser extent, GM3 and SM were significantly downregulated in eGDM. HexCer-NS(d40:2) emerged as having the strongest negative association with eGDM risk in our models, suggesting that specific glycosphingolipids are reduced in states of metabolic stress. Inverse associations between HexCer and SM species have previously been observed in early pregnancy among women who later developed GDM^13^; here, we show that these differences are already evident at the time of eGDM diagnosis, suggesting that variations in sphingolipid metabolism may contribute to glycemic dysregulation much earlier in gestation than previously recognized. Together, these opposing patterns likely reflect impaired sphingolipid remodeling and broad dysregulation of the sphingolipid axis in eGDM, marked by accumulation of bioactive ceramides and loss of potentially regulatory glycosphingolipids.

The present study has several strengths. To our knowledge, this is the first study to examine the plasma lipidome in women diagnosed with eGDM – a subgroup with pathophysiological distinctions from later-onset GDM, yet uncharacterized in lipidomic studies to date. By leveraging highly sensitive LC/MS and profiling women at the time of eGDM diagnosis using contemporaneous OGTT-based criteria, we add an important and previously undefined molecular dimension to the understanding of eGDM. We applied stringent quality control thresholds, excluding lipids with high technical variability or missing values, thereby maintaining high quality and reproducibility in our analyses. We conducted detailed statistical analyses demonstrating that the identified lipids were robustly associated with eGDM, using multiple levels of data analysis, from exploratory visualization of data structure using PCA, correlation analysis, and k-means clustering to more rigorous modeling and network-based clustering approaches, such as linear regression and WGCNA. These complementary statistical frameworks, including both class-level and individual species-level approaches, enabled identification of consistent lipid patterns that were both statistically robust and mechanistically meaningful, aligning with known metabolic pathways. Functional annotation through lipid ontology enrichment further supported the biological plausibility of these patterns. Our ethnically diverse and clinically well-characterized cohort was derived from TOBOGM, the largest randomized clinical trial of women with eGDM. This enabled detailed multivariable analysis, demonstrating that the observed lipid associations were independent of key risk factors, including maternal age, ethnicity, BMI, education and neonatal sex. These covariates are clinically relevant, with previous studies reporting ethnic differences in GDM prevalence^69^, and higher risks in women with lower educational attainment^70^ and in those carrying a male fetus^71^.

Limitations include that, although lipidomics in eGDM have not been previously characterized, our sample size is modest and limited our ability to investigate lipidomic variations within different ethnic or BMI subgroups. It was also not possible to explore the heterogeneity of eGDM as reported previously^72,73^, including those who regressed to normal glucose tolerance and those who only developed later GDM. The cross-sectional design also precluded inference about causality or temporal lipid changes across gestation. Furthermore, while untargeted lipidomics offers broad coverage, accurate identification of hundreds of lipids can be challenging and often lacks complete structural resolution, with some lipid classes underrepresented as seen in the limited FAs captured here. Here, FAs were measured in the form of gas-phase dimers rather than canonical deprotonated anions, due to an unintended misassignment issue a problem only recently recognized with MS/MS based identification^74^. Although measuring FAs as dimers is uncommon, the measurements correlate extremely strongly with the corresponding deprotonated anion (R squared >0.98 in all cases, Supplementary Data 6) and are unlikely to materially affect the findings. Moreover, the finding of elevated FA and DAG relative to TAG could reflect post-sampling lipolysis. While we did not quantify glycerol to confirm this, standardized sample collection and rapid processing make artifactual associations unlikely, and QC analyses demonstrated reproducibility across lipid classes. Moreover, circulating lipids may not fully reflect lipid dynamics in metabolically active tissues relevant to GDM pathophysiology, such as the placenta, skeletal muscle or adipose tissue. Although we adjusted for key clinical covariates, residual confounding cannot be entirely excluded, particularly since dietary intake and other environmental exposures which can influence lipid metabolism, were not assessed. Attenuation after adjustment suggests that underlying risk factors partly explain the observed lipid differences; however, the persistence of several lipid species across models indicates that these alterations are not fully accounted for by these factors. Further prospective studies incorporating larger, longitudinally profiled populations with diverse risk profiles are now needed to validate the lipidomic signature identified here and to evaluate its utility for early identification and risk factor-based screening and stratification.

## Conclusions

In summary, this study identifies a distinct lipidomic signature of eGDM, comprising elevated concentrations of glycerolipids (DAG), FA, and ethanolamine-containing glycerophospholipids (PE and LPE), alongside downregulation of glycosphingolipids (HexCer) and choline-containing glycerophospholipids (LPC and PCe). FA and DAG emerged as the strongest and most consistent lipids associated with eGDM and dysglycemia, independent of clinical risk factors, and were enriched in pathways related to lipid storage, membrane remodeling, and signaling. Our findings offer important molecular insights into the pathophysiology of eGDM and identify candidate lipid species for further investigation and validation in this context.

## Supplementary information

Supplementary Information

Description for Supplementary Data Files

Supplementary Data 1

Supplementary Data 2

Supplementary Data 3

Supplementary Data 4

Supplementary Data 5

Supplementary Data 6

## Data Availability

All source data and generated data supporting the findings of this study and figures are available as supplemental data files and are publicly available on Monash Bridges (DOI: 10.26180/31850968). Individual participant-level data underlying this article cannot be shared publicly due to participant privacy protection and ethical restrictions. Deidentified participant data may be obtained from the corresponding author (aya.mousa@monash.edu) upon reasonable request and subject to appropriate approvals including via the TOBOGM Core Investigator Group. Requests will be reviewed and responded to within 2–4 weeks and, if approved, data will be made available under a data use agreement that specifies restrictions on use, including limitations on re-use, sharing, and adherence to approved study purposes.

## Code availability

No new code was developed for this study, and only existing general pipelines were used to generate the results. However, any code used for the data analysis is available from the first author (saeede.saadati@monash.edu) or the corresponding author (aya.mousa@monash.edu) upon reasonable request.
