# Supplementary material for: Lipidomic profiling reveals a distinct lipidomic signature of early gestational diabetes
Source: Commun Med (Lond). 2026 Jul 4;6:562. doi: 10.1038/s43856-026-01765-6 (PMC13714274; doi:10.1038/s43856-026-01765-6)
Supplement: Supplementary file 1 [file 43856_2026_1765_MOESM1_ESM.pdf]

Supplementary Information

1. Associations between lipids with demographic and clinical variables overall and within early and non-GDM groups

Correlation and linear regression analyses were applied to examine associations between lipids and demographic and clinical variables, with results provided in Supplementary Data 2 and 3, and visualized in Supplementary Fig. 1a-f.

On correlation analysis, FA showed the strongest positive correlation with BMI across the cohort (Supplementary Data 2). This was followed by SM, Cer and LPC, which also correlated positively with BMI and were the lipid classes most strongly associated with White ethnicity (r = 0.20 - 0.33). White ethnicity also showed weaker positive correlations with LPE, LPI and cholesterol esters (CE) (r = 0.16 – 0.19), and a negative correlation with PE (r = -0.17). For East and South East Asian ethnicities, the strongest positive correlations were seen with ether-linked phospholipids, PCe and PEe, whereas South Asian ethnicity showed strong negative correlations with these same lipids (r = -0.40 – -0.44), and weaker negative correlations with FA, Cer and LPC (r = -0.17 – -0.19). PCe and PEe, and to a lesser extent DAG, also showed positive correlations with maternal age.

Linear regression modelling was applied using maternal age, BMI, and gestational week at sampling as predictors, with adjustment for gravidity, ethnicity, and family history of diabetes. Results obtained from each model are presented in Supplementary Data 3. There were no significant associations between lipidomic profiles and maternal age in both early GDM and non-GDM groups (Supplementary Fig. 1a). FA, "other", and SM lipid classes were positively associated with BMI exclusively in the non-GDM group (Supplementary Fig. 1b). Analyses of lipid classes and BMI revealed stronger associations in the non-GDM group, particularly for

the FA and SM classes (Supplementary Fig. 1d). The density of data on scatter plots suggested that early GDM participants exhibited steady or slightly higher lipid class levels across different BMI ranges, indicating relative independence from variations in BMI (Supplementary Fig. 1d).

PE and PS lipid classes were significantly associated with gestational week at sampling among both the non-GDM and early GDM groups, whereas the TAG class showed significant associations only in the non-GDM group (Supplementary Fig. 1c). Analyses of lipid classes and gestational week at sampling showed stronger associations in non-GDM participants compared to participants with early-GDM (Supplementary Fig. 1e). These results indicate that variations in some lipid classes in the early GDM group (FA, SM and TAG) are relatively independent of variations in BMI and gestational week at sampling.

Next, we explored how specific lipid species were associated with variations in BMI and gestational week at sampling within the non-GDM and early GDM groups. We performed similar linear modelling analyses at the lipid species level for both groups to identify significantly associated species (adjusted $P \leq 0.05$; Supplementary Data 3). We then determined the intersection of lipid species that were significant in at least one group. Supplementary Fig. 1f illustrates the lipid species significantly associated with BMI and gestational week at sampling in at least one of the non-GDM or early GDM groups. We found lipid species from seven classes associated with variations in BMI, of which six classes showed positive associations in the non-GDM group, while showing little to no association or negative associations in the early GDM group. For example, almost all species in the SM class were positively associated with BMI in the non-GDM group, while only four species showed positive and two showed negative associations in the early GDM group. Lipid species from the PE, PC, PCe classes were negatively associated with BMI in the early GDM group, while showing no association or positive associations in the non-GDM group (Supplementary Fig.

1f). Lipid species belonging to FA were positively associated with BMI in the non-GDM group but showed no association in the early GDM group.

PE and TAG classes were associated with gestational week at sampling primarily within the non-GDM group, while only a few were associated within the early GDM group (Supplementary Fig. 1f). These findings suggest that the lipidomic profile in non-GDM participants differs significantly with variations in BMI and gestational week at sampling, whereas potential physiological and metabolic alterations in participants with early GDM might obscure these changes.

Altogether, we identified significant associations between the lipidomic profile, at both the class and species levels, and variations in BMI and gestational week at sampling. The results indicate that lipidomic profiles in early GDM differ to those in non-GDM by variations in BMI and gestational week at sampling, with a higher rate of variability in the non-GDM group.

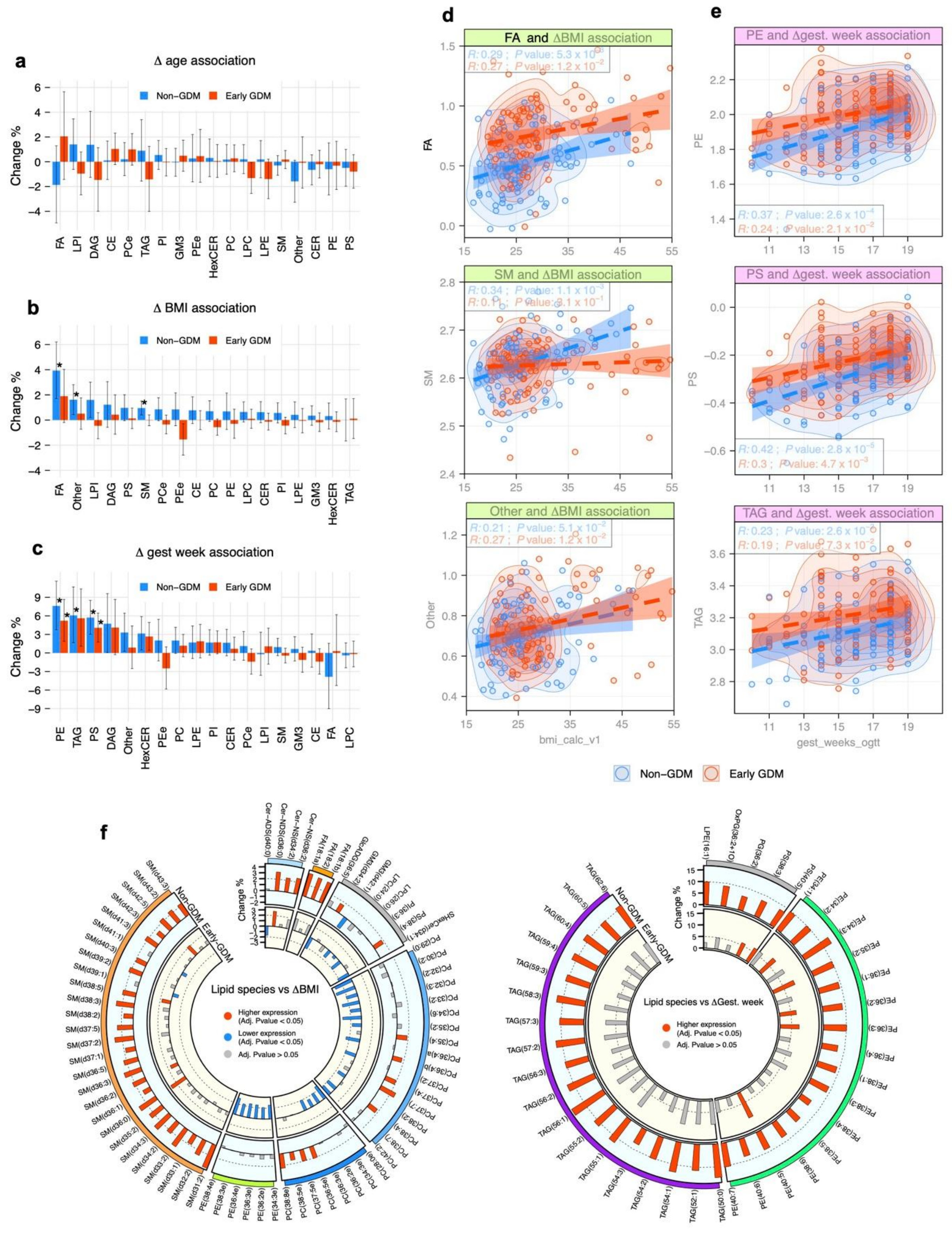

Supplementary Figure 1. Association of lipidomic profile with maternal age, BMI, and gestational week at sampling.

(a-c) Linear modeling was performed to assess associations between lipid classes and maternal age, BMI, and gestational week at sampling, analyzed separately for non-GDM and eGDM groups to uncover group-specific associations. The error bars represent ± S.D., and a star symbol denotes significance (adjusted P < 0.05). **(d-e)** Correlation plots illustrate the relationships between lipid classes and BMI or gestational week at sampling. Blue and red represent correlations within non-GDM and eGDM groups, respectively. Shaded areas around the fitted lines indicate confidence intervals and topographical regions represent sample density within the scatterplot. **(f)** Circular bar charts highlight lipid species significantly associated (adjusted $P < 0.05$) with BMI and gestational week at sampling based on linear modeling. The charts represent the union of significant lipids across non-GDM and eGDM groups, illustrating lipid concentration variations specific to each group. Lipids are categorized by class, with values indicating percentage variation.

All regression models were adjusted for gravidity, ethnicity, and family history of diabetes, as well as for maternal age, BMI, and gestational age at sampling when these variables were not the primary predictors. Maternal age, BMI, and gestational week at sampling were treated as continuous variables.

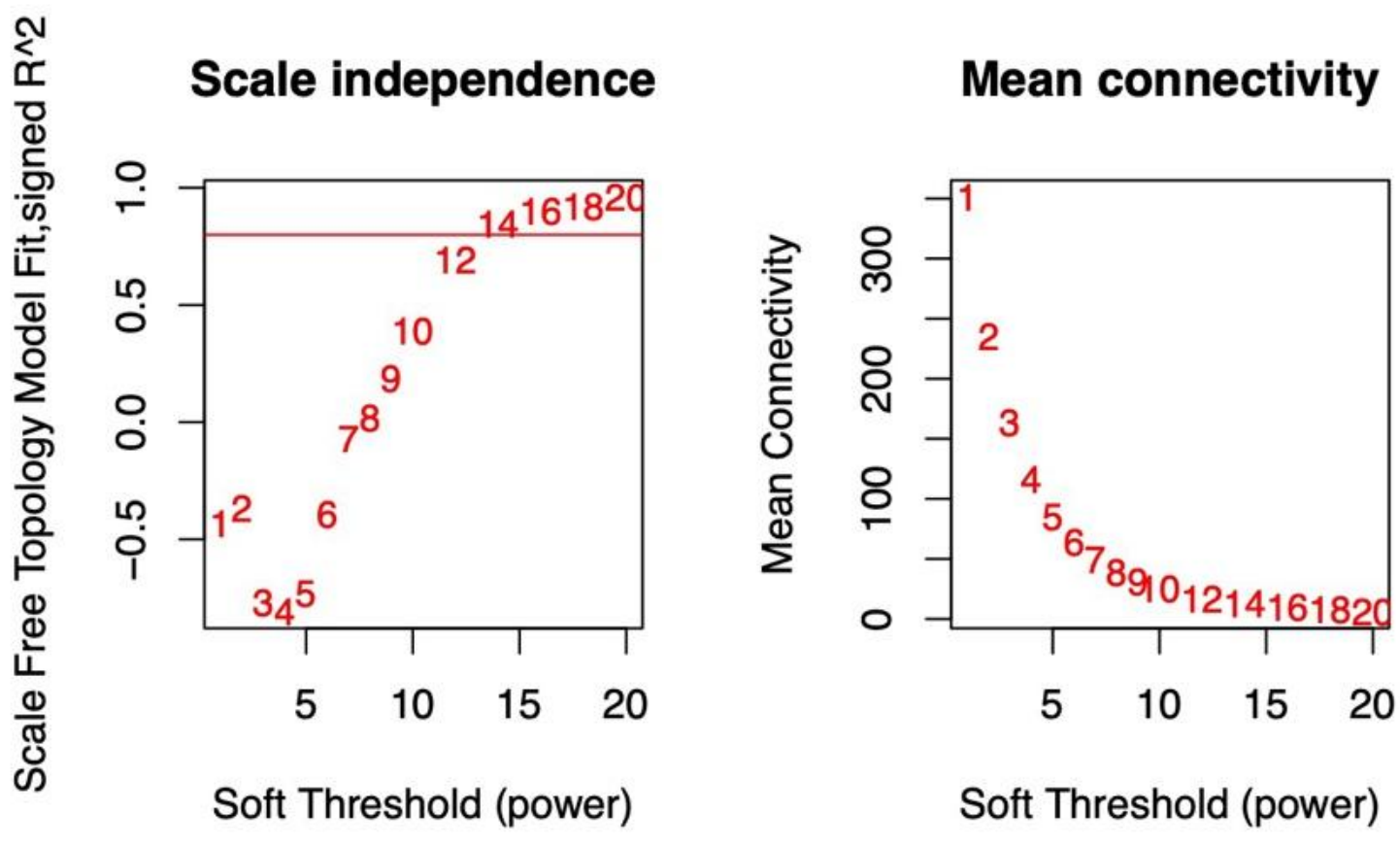

Supplementary Figure 2. Results of the scale-free topology model fit analysis for WGCNA. The plots show the scale independence and mean connectivity across different soft-thresholding powers.
