# Supplementary material for: Lipidomic profiling reveals a distinct lipidomic signature of early gestational diabetes
Source: Commun Med (Lond). 2026 Jul 4;6:562. doi: 10.1038/s43856-026-01765-6 (PMC13714274; doi:10.1038/s43856-026-01765-6)
Supplement: Supplementary file 2 [file 43856_2026_1765_MOESM2_ESM.docx]

**Descriptions for Supplementary Data Files**

**Supplementary Information:** Summary of the results of correlation analyses between lipid classes and eGDM.

**Supplementary Data 1:** Detailed information on lipid quantification and classification used in the study. S1: List of lipid species within each class; S2: Lipid species concentrations across study groups; and S3: Number of lipid species in each class; and S4: Median CV for lipid species.

**Supplementary Data 2:** Results of PCA, lipid class comparisons, and correlation analyses across study groups. S1: Eigenvector values of each point across principal components; S2: Lipid class concentration across study groups; S3: Comparison of Log10 transformed of lipid class concentrations in each group of participants; and S4: Correlation analyses between lipid classes and eGDM and clinical risk factors for GDM in each group.

**Supplementary Data 3:** Linear regression analyses of lipid class associations with BMI change and gestational week change in eGDM and non-GDM groups. S1: Linear regression analysis of lipid class of lipidomic profile of control participants (non-GDM) versus BMI change; S2: Linear regression analysis of lipid class of lipidomic profile of participants with early-GDM versus BMI change.; S3: Linear regression analysis of lipid class of lipidomic profile of control participants (non-GDM) versus gestational week change; and S4: Linear regression analysis of lipid class of lipidomic profile of participants with early-GDM versus gestational week change.

**Supplementary Data 4:** Linear regression analysis of differential lipid species by eGDM status. S1: Model 1 (unadjusted model); S2: Model 2 (adjusted for maternal age, BMI, gravidity, gestational week at sampling, ethnicity, and family history of diabetes); S3: Model 3 (Model 2 plus adjustment for educational qualification); S4: Model 4 (Model 3 plus adjustment for neonatal sex); S5: Model 5 (Model 2 plus adjustment for PCOS, previous GDM and systolic blood pressure (SBP)); and S6: The common significant lipid species across all linear regression models.

**Supplementary Data 5:** Lipid clusters associated with eGDM using WGCNA. S1: modules (sub-networks) associated with clinical data of participants; S2: Information of modules identified by WGCNA; and S3: WGCNA LION.

**Supplementary Data 6:** Correlation between gas-phase dimer and deprotonated anion measurements of FAs.
